# Eltrombopag for Adults and Children with Immune-Refractory Thrombocytopenic Purpura: A Systematic Review

**DOI:** 10.3390/jcm12123872

**Published:** 2023-06-06

**Authors:** Danielle Francisco Honorato de Barros Torelli, Crystian Bitencourt Soares Oliveira, Gisele Alborghetti Nai, Evelinda Marramon Trindade, Luiz Euribel Prestes-Carneiro

**Affiliations:** 1 Master’s Program in Health Sciences, Oeste Paulista University, Presidente Prudente 19050-920, SP, Brazilcrystian@unoeste.br (C.B.S.O.);; 2 Health Technology Assessment Center (NATS), Oeste Paulista University, Presidente Prudente 19050-920, SP, Brazil; 3 Pediatrics Department and Emergency Department, Regional Hospital of Presidente Prudente, Oeste Paulista University, Presidente Prudente 19050-920, SP, Brazil; 4 Health Technology Assessment Center (NATS) of the São Paulo University Medical School, Clinics Hospital, São Paulo 01246-903, SP, Brazil; 5 São Paulo State Health Secretary, São Paulo 05403-000, SP, Brazil

**Keywords:** immune thrombocytopenic purpura, erythropoietin receptors, systematic review, blood coagulation, hematologic drugs

## Abstract

Eltrombopag is an agonist that binds to the membrane-bound domain of the thrombopoietin receptor used in immune thrombocytopenic purpura (ITP). We conducted a meta-analysis of randomized controlled trials to assess the efficacy and safety of eltrombopag in adults and children with refractory ITP. Adults who received eltrombopag had a significantly better platelet response (relative risk [RR], 3.65; 95% confidence interval [CI], 2.39–5.55), but there were no differences in the incidence of bleeding (RR, 0.8; 95% CI, 0.52–1.22) and adverse effects (RR, 0.99; 95% CI, 0.55–1.78) compared with the placebo. In children, there was no difference between eltrombopag and placebo for a platelet response >50,000/mm^3^ (RR, 3.93; 95% CI, 0.56–27.79) and the number of adverse events (RR, 0.99; 95% CI, 0.25–1.49); however, a lower incidence of bleeding was observed (RR, 0.47; 95% CI, 0.27–0.83). Treatment with eltrombopag protected adults and children from severe disease and death.

## 1. Introduction

Immune thrombocytopenic purpura (ITP) is an autoimmune disorder characterized by a reduction in the number of circulating platelets (<100 × 10^3^/mm^3^), leading to petechiae, purpura, severe bleeding episodes, and death [1]. Triggered by a deregulation of the immune system, about one-third of newly diagnosed patients do not have bleeding, only thrombocytopenia [2]. Usually, IgG autoantibodies adhere to platelet membrane antigens and are recognized by spleen macrophages and other areas of reticuloendothelial tissue. Platelets are destroyed, leading to a shorter average lifespan of platelets and, consequently, lower platelet counts in peripheral blood [3]. The diagnosis of ITP is based primarily on exclusion of other causes of thrombocytopenia using the patient’s history, physical examination, complete blood counts, and peripheral blood smear evaluation (to exclude other hematologic conditions, including hereditary thrombocytopenia and pseudothrombocytopenia) [4]. ITP is classified as primary (80%) or secondary (20%) and by disease duration as newly diagnosed (0–3 months), persistent (>3–12 months), or chronic (>12 months) [4]. It is usually self-limiting in children, progressing to spontaneous remission; in contrast, spontaneous remission occurs in <10% of adults [5].

Treatment focuses on increasing the platelet count to a safe level to prevent severe bleeding and reduce the incidence of death [3,6,7]. Drug treatment is indicated for patients with severe thrombocytopenia (<20 × 10^3^/mm^3^) or those with bleeding associated with thrombocytopenia (<50 × 10^3^/mm^3^). First-line therapies include corticosteroids, intravenous immunoglobulin (IVIG), or anti-D immunoglobulin [6]. Second-line therapies are indicated in refractory cases, that is, those with persistent thrombocytopenia even after the use of first-line drugs. Furthermore, for patients who relapse or fail after splenectomy, the use of thrombopoietin receptor agonists is indicated [3,5,6]. Eltrombopag is an agonist that binds to the juxtamembrane domain of the thrombopoietin (TPO) receptor, resulting in signaling through the JAK/STAT, AKT, and MAPK pathways. It is structurally different from endogenous TPO, with a non-competitive mechanism activating TPO receptors, resulting in megakaryocyte proliferation and differentiation, and leading to increased platelet production. Eltrombopag binds to a site other than TPO in c-mpl, with additive effects. After oral administration, eltrombopag is excreted in the feces, suggesting that the liver is the main organ of elimination. However, the effectiveness of eltrombopag in children and adults is not completely understood, and side effects have been reported. Some studies in children, such as PETIT [7] and PETIT2 [8], reported that the most common adverse effects of eltrombopag therapy were headache, upper respiratory tract infection, nasopharyngitis, diarrhea, and changes in transaminases. Although the use of eltrombopag has also been associated with an increased risk of thrombosis, it is difficult to accurately determine if eltrombopag therapy was the cause, especially in adults. Thus, the safety of eltrombopag in children and adults needs to be better understood [9,10,11,12]. The use of eltrombopag was approved by the US Food and Drug Administration in adults in 2008 and in children > 1 year of age in 2015 as a second-line therapy [10,11,12]. In 2016, the European Commission also approved the use of eltrombopag. In Brazil, its use is authorized for children > 6 years of age. Previous reviews have investigated the effectiveness of eltrombopag with refractory ITP: Massaro et al. [6] investigated children and Wang et al. [13], Elgebaly et al. [14], and Kolanis et al. [15] published reports on a mix of children and adults. However, these reviews have some methodologic flaws, including the lack of registration, lack of an appropriate assessment of the overall quality of evidence, or the lack of assessment of statistical heterogeneity for the meta-analysis. Moreover, studies that include adults and children in the same analyses make it difficult to estimate the effectiveness of eltrombopag; thus, there is a need for additional studies that consider specific age groups.

Internationally, the incidence of ITP is 1.6–2.7 cases per 100,000 adults, increasing with age [1,3]. There are no publications regarding its incidence and prevalence in the Brazilian population so far. In 2018, the Brazilian National Commission for the Incorporation of Technologies, CONITEC, included eltrombopag in the public Unified Health System (SUS). However, since then, although, in theory, eltrombopag should be available free of charge throughout the country, not all local managers have listed it. Thus, many patients with ITP need to go through the Courts to obtain access, delaying the treatment. In Brazil, eltrombopag is considered to be an important therapeutic tool for ITP. However, so far, there are no Brazilian data regarding its effectiveness, safety, and how many people are using or have used eltrombopag. Therefore, we aim to update the available evidence on the efficacy of eltrombopag and its safety in children and adults with refractory ITP stratified by age group, with regard to the lasting response, occurrence of adverse events, platelet response, use of rescue therapy, and incidence of bleeding.

## 2. Methodology

The protocol and this review follow the PRISMA-P (Preferred Reporting Items for Systematic review and Meta-Analysis Protocols) checklist [16]. The protocol of this review was registered in the Open Science Framework database (https://osf.io/x8ce3 (accessed on 21 November 2022)).

### 2.1. Search Strategy

The guiding question for the search was: How effective is eltrombopag in adults and children with refractory ITP compared with placebo?

From inception to 21 January 2023, a detailed and automated search was performed across the following electronic databases: Medline (by Ovid), Embase, Cochrane Collaboration, Web of Science, Clinicaltrials.gov (https://clinicaltrials.gov/ (accessed on 21 January 2023)), and the World Health Organization registry database (https://trialsearch.who.int/ (accessed on 21 January 2023)). There were no restrictions regarding the date and language of publication. A combination of descriptors related to randomized clinical trials, ITP, and eltrombopag was used for the search strategy.

### 2.2. Selection of Studies

Titles and abstracts were evaluated by two independent reviewers. (D.F.H.B.T. and C.B.O.). Disagreements were resolved by consensus between the two researchers and in discussion with the reviewers. The full texts of the studies selected according to review of the titles and abstracts were evaluated considering the inclusion criteria of this review. Randomized clinical trials investigating the efficacy and safety of eltrombopag in adults and children with refractory ITP compared with placebo were selected. Refractory ITP was defined as persistence of thrombocytopenia and a lack of response to first- and second-line treatments. The selected population comprised patients with primary ITP. ITP is an autoimmune disorder characterized by reduced platelet counts, <100 × 10^3^/mm^3^, and increased risk of bleeding in the absence of another cause or disorder associated with thrombocytopenia [17]. Secondary ITP was excluded (e.g., thrombocytopenia triggered by drugs or other diseases such as Evans syndrome, primary immunodeficiency due to underlying autoimmune infection, hepatitis C, or others).

The World Health Organization (WHO) has standardized the classification scale to measure the severity of bleeding. The scale ranges from grade 0 to 4; grade 3 applies when the patient has gross blood loss that requires transfusion, and grade 4 applies when there is debilitating blood loss, retinal or cerebral, associated with fatality [18].

The primary endpoints for this review were a durable platelet response (i.e., platelet count ≥ 50 × 10^3^/mm^3^ for 4 or more weeks, consequently reducing the chances of clinically significant bleeding) and adverse events. Secondary endpoints were overall platelet response (achieving at least one platelet response ≥ 50 × 10^3^/mm^3^ during treatment), use of rescue medication (patients receiving any unscheduled treatment or new treatment), and the incidence of clinically active bleeding [18].

### 2.3. Data Extraction

Two independent evaluators (D.F.H.B.T. and C.B.O.) used a standardized form to extract information on the intervention and control groups regarding sample size, age, sex, measures related to chosen outcomes, and clinically relevant characteristics. Outcomes were recorded as the number of people reaching a given outcome in the follow-up assessment from the total number of participants in each group.

### 2.4. Risk of Bias Assessment

Two independent evaluators (D.F.H.B.T. and C.B.O.) assessed the methodologic quality and risk of bias of the selected studies using the Cochrane Risk of Bias (RoB) tool [19] for randomized clinical trials. The RoB tool includes the following domains:

1. Generation of randomization sequence: evaluates the methods used to allocate participants in groups, such as random tables, software, and others.
2. Allocation secrecy: evaluates the methods used to ensure the implementation of the generated randomization sequence, with telephone exchange, virtual platforms, or others.
3. Blinding of the participants and the team: evaluates the methods used to conceal to which group the participants were allocated from other participants and from the care team.
4. Blinding of outcome assessors: assesses the methods used to ensure that outcome assessors do not know to which group the participants were allocated.
5. Incomplete outcome data: assesses the impact of loss of participants on the results throughout the study.
6. Selective reporting of outcomes: evaluates the alignment between the outcomes planned in the study protocol and the outcomes assessed and/or reported.
7. Other sources of bias: to ascertain any other source of bias not considered in the previously described domains, such as imbalance between the groups compared at the study baseline, early interruption of the study, or others.

Any disagreement between the evaluators was resolved by discussion until consensus was reached. The domains were rated as high, low, or uncertain risk of bias, considering the assessment for each item. Then, the studies were rated as having high, moderate, or low risk of bias, as follows:

- Low risk of bias: if all domains were judged to have a low risk of bias;
- Some concerns: if at least one domain was judged to raise some concerns and no domain was judged to have a high risk of bias;
- High risk of bias: if at least one domain was judged as having a high risk of bias or if multiple domains were judged to have some concerns, so that confidence in the outcome was substantially reduced.

### 2.5. Data Analysis and Synthesis of Results

The total sample was extracted for each group as well as the number of people with a lasting response, adverse events, platelet response, use of rescue therapy, and incidence of bleeding. Heterogeneity was assessed using I^2^; I^2^ > 50% was considered substantial heterogeneity. Random effect models were used to obtain relative risk (RR) and the respective 95% confidence intervals (CIs). The analyses were performed using Review Manager (version 5.4).

The overall strength of the evidence was assessed using the Grades of Recommendation, Assessment, Development, and Evaluation (GRADE) approach [20]. The following domains were evaluated: study design (randomized clinical trial), risk of bias (significant when >25% of studies included in the analysis came from trials with a high risk of bias), inconsistency (when heterogeneity between studies was large (I^2^ > 50%)) and imprecision (when the total number of events was <300 for each result). The overall strength of evidence was rated from very low to high; high-quality evidence (low RoB) generated greater confidence in the meta-analysis results.

## 3. Results

**Figure 1:**
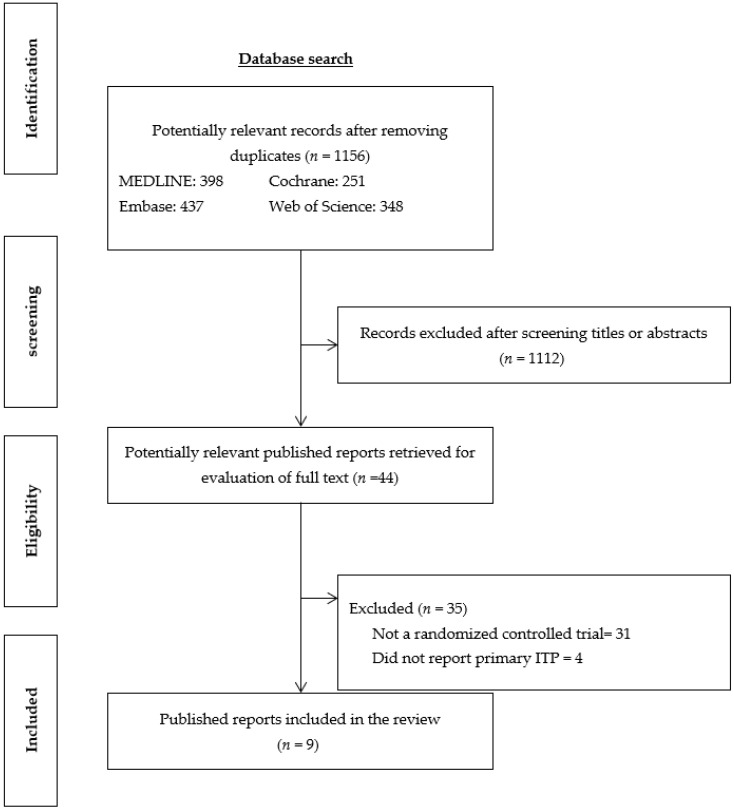
Flowchart of the studies included in the systematic review. ITP, immune thrombocytopenic purpura.

The searches in the electronic databases found 1156 studies after removing duplicates. Of these, 1112 were excluded after assessing the titles and abstracts because they did not meet the inclusion criteria for this review. Therefore, 44 full texts were selected for evaluation. After reviewing the full texts, a further 35 studies were excluded because they were not randomized clinical trials (*n* = 31) or did not include participants with primary ITP (*n* = 4). Finally, nine studies [7,8,21,22,23,24,25,26,27] from the published literature indexed in the bibliographic databases were considered eligible for this systematic review. None of the studies indexed at the clinical trial registry databases searched were considered eligible. The PRISMA flow diagram of the identification and selection process is shown in Figure 1.

The studies were conducted in China (*n* = 2), Japan (*n* = 1), or were multicenter studies in several countries (*n* = 6). Most studies recruited adults (*n* = 7); only two studies included children with ITP. In total, 915 individuals with primary ITP were included in the nine studies: 639 adults and 276 children. The median age of the adult participants ranged from 40.9 years to 60.5 years; 249 participants were male and 506 were female. The median age of the children ranged from 9 years to 10 years; 75 of the children were male and 84 were female. The dose of eltrombopag used in adults ranged from 12.5 mg/day to 75 mg/day, and treatment duration ranged from 8 to 24 weeks. In children, the dose varied by age, ranging from 0.8 mg/kg/day to 1.5 mg/kg/day for children aged 1–5 years, from 12.5 mg/day to 50 mg/day for children aged 6–11 years, and from 25 to 50 mg/day for children aged 12–17 years. The duration of treatment was 24–28 weeks in all age groups. All studies compared eltrombopag with a placebo. The characteristics of the studies are presented in the Supplemental Material (Table S1).

**Figure 2:**
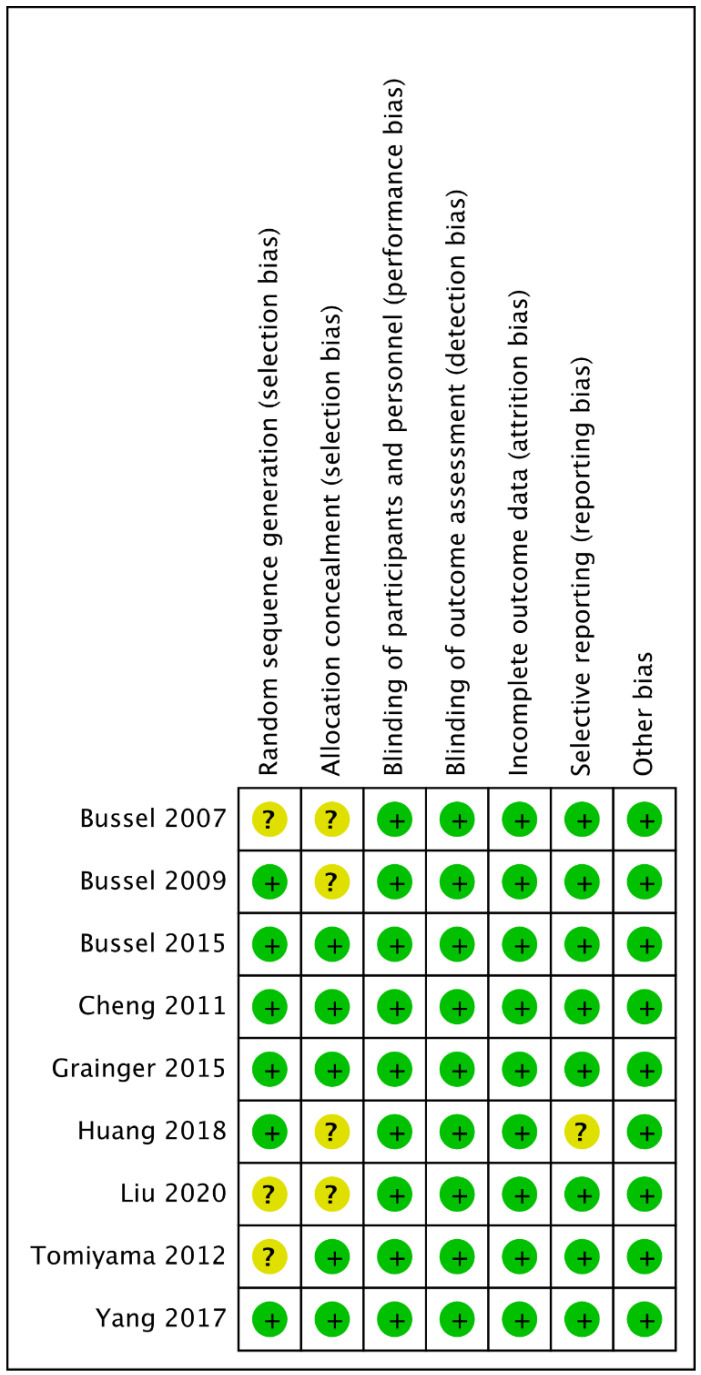
Risk of bias summary. The authors’ judgment for each study. Green, low risk of bias; yellow, uncertain risk [7,8,21,22,23,24,25,26,27].

Figure 2 shows the risk of bias for the studies included in this systematic review. All studies had a low risk of bias for the blinding of participants, blinding of results, blinding of outcome assessors, and selective reporting of outcomes. Three studies did not have sufficient information on the overall randomization sequence and secret allocation, or on selective reporting in one study. The studies were rated as having low [8,21,22,25] or moderate [22,23,24,25,26,27] risk of bias.

### 3.1. Analysis of Primary Outcomes

**Figure 3:**
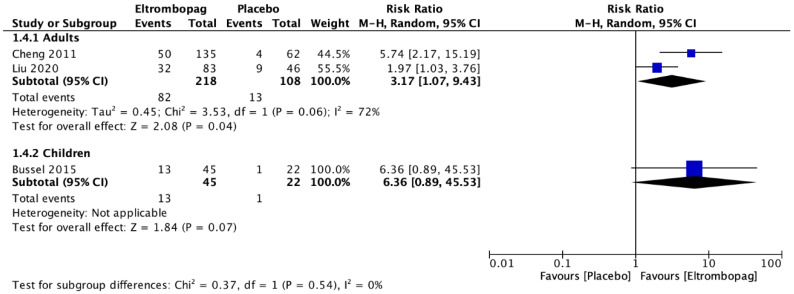
Comparison of the lasting response with eltrombopag and placebo in adults and children with immune thrombocytopenic purpura [7,21,24].

**Figure 4:**
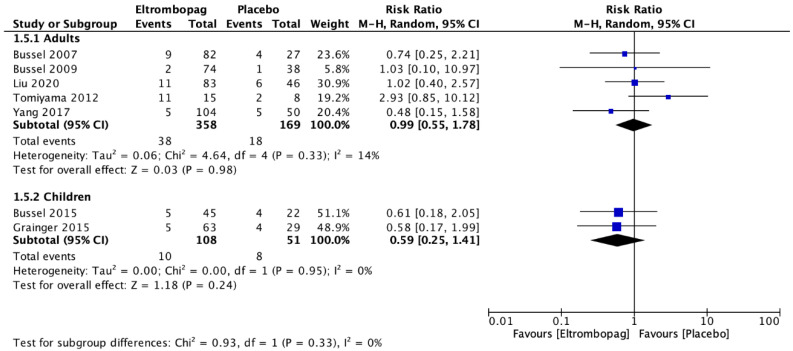
Comparison between the occurrence of adverse events with eltrombopag and placebo in adults and children with immune thrombocytopenic purpura [7,8,22,23,24,25,26].

In total, seven studies including 759 participants reported the results for adults with ITP [7,8,21,22,23,24,25]. The meta-analysis demonstrated that adults who received eltrombopag had a significant lasting response (two studies; *n* = 326; RR, 3.17; 95% CI, 1.07–9.43; but with low certainty of evidence) (Figure 3). In addition, there was no difference between the groups on the occurrence of adverse events (five studies, *n* = 527; RR, 0.99; 95% CI, 0.55–1.78; moderate certainty of evidence) in comparison with the placebo group (Figure 4). Two studies [21,23] that included 159 children with ITP reported the results comparing eltrombopag with placebo groups. The meta-analysis estimated that there was no difference between eltrombopag and placebo for lasting response (one study; *n* = 67; RR, 6.36; 95% CI, 0.89–45.53; moderate certainty of evidence) (Figure 3) and the number of adverse events (two studies; *n* = 159; RR, 0.99; 95% CI, 0.25–1.4; moderate certainty of evidence) (Figure 4).

### 3.2. Analysis of Secondary Outcomes

**Figure 5:**
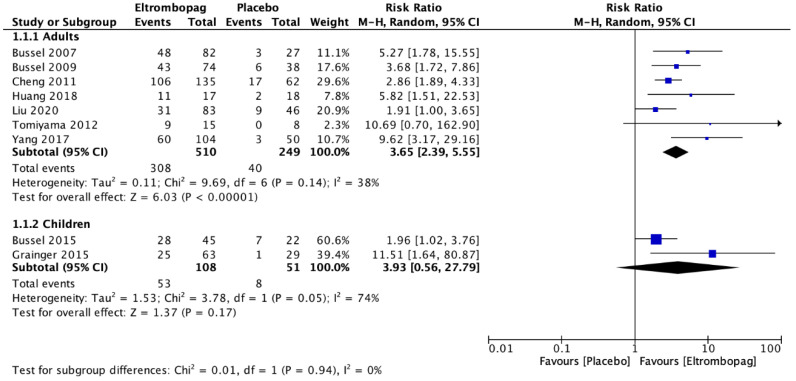
Comparison between the platelet response with eltrombopag and placebo in adults and children with immune thrombocytopenic purpura [7,8,21,22,23,24,25,26,27].

**Figure 6:**
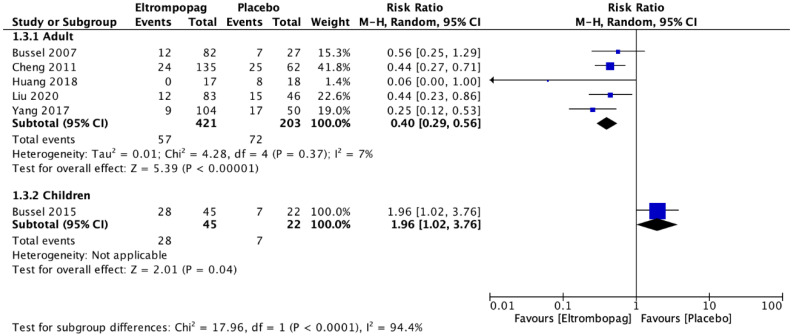
Comparison between the use of eltrombopag rescue therapy and placebo in adults and children with immune thrombocytopenic purpura [7,21,23,24,25,27].

**Figure 7:**
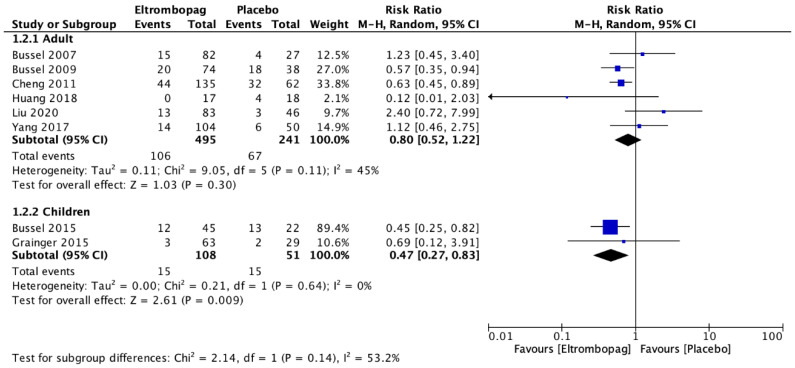
Comparison between the occurrence of bleeding with eltrombopag and placebo in adults and children with immune thrombocytopenic purpura [7,8,21,22,23,24,25,26].

Adults who received eltrombopag had a significant platelet response >50,000/mm^3^ compared with the placebo group (seven studies; *n* = 759; RR, 3.65; 95% CI, 2.39–5.55) (Figure 5). Significantly less rescue therapy was used in those patients who received eltrombopag (five studies; *n* = 624; RR, 0.4; 95% CI, 0.29–0.56) compared with patients who received a placebo (Figure 6). However, there was no difference between the groups for the occurrence of bleeding (six studies; *n* = 736; RR, 0.8; 95% CI, 0.52–1.22) (Figure 7).

The meta-analysis revealed that there was no difference in children between the eltrombopag and placebo groups in platelet response >50,000/mm^3^ (two studies; *n* = 159; RR, 3.93; 95% CI, 0.56–27.79) (Figure 5). Children who received eltrombopag used more rescue therapy (one study; *n* = 67; RR, 1.96; 95% CI, 1.02–3.76) compared with those who received placebo (Figure 6). However, there was a significant difference between the groups regarding the occurrence of bleeding; significantly more bleeding events occurred in the group that received placebo (two studies; *n* = 159; RR, 0.47; 95% CI, 0.27–0.83) (Figure 7).

The certainty of evidence for the outcome of platelet counts > 50,000/mm^3^ was high in adults and low in children. The certainty of evidence for analysis of bleeding events and the use of rescue therapy was moderate (due to serious imprecision) for both adults and children [20].

The quality of the published evidence for the use of eltrombopag compared with placebo in adults and children with ITP is shown in Table 1.

## 4. Discussion

One of the main goals and the originality of this meta-analysis is the age group stratification. Results for adults and children have not been shown separately in the previous literature on ITP.

Treatment with eltrombopag is highly effective in adults, with greater platelet counts and a lasting response, and fewer requirements for rescue therapy compared with placebo. In addition, there was no difference between the groups for the occurrence of bleeding or adverse events. In contrast, children who received eltrombopag did not have a greater platelet response, did not have more chance of having a lasting platelet response, or did not use less rescue therapy; they did not have fewer adverse events. However, the occurrence of bleeding was lower in children compared with the placebo groups, although the evidence varied between low and high quality. These results are in line with the published data on the efficacy and safety of eltrombopag in patients with ITP. Ahmed et al. [28] and Elgebaly et al. [14] investigated the effects of eltrombopag in adults and children together in the same analysis. They demonstrated significant results in global platelet response, use of rescue therapy, and the incidence of any or significant bleeding. In addition, as in our results, there was no significant difference in total adverse events compared with the placebo. Another meta-analysis investigated eltrombopag and romiplostim in the same sample only with children [6]. Massaro et al. [6] found a greater platelet response, in contrast to the result from our analysis. However, when they analyzed the specific subgroup who received eltrombopag alone [6], they demonstrated a lower incidence of bleeding, as in our results. Although most of our analysis of outcomes reiterates the findings of previously published literature, some results were conflicting. For example, the benefit of a long-lasting platelet response found in adults was evident in the analyses stratified by age group but was not seen in the previous aggregated groups [14,28]. Another difference was found in the comparator group in the previously published results on children [6]. Here, eltrombopag was compared with placebo because romiplostim was denied listing by CONITEC.

ITP is a hematologic disease that is characterized by thrombocytopenia due to the destruction of platelets by autoantibodies. This favors bleeding, which interferes with the form of treatment and the quality of life of patients. In adults, the use of eltrombopag has been shown to reduce the incidence of bleeding and allow for a better global platelet response. The incidence of bleeding is related to the platelet count; the highest incidence of severe bleeding occurs in patients with a platelet count < 20,000/mm^3^. This explains why adults with higher platelet counts have a lower incidence of bleeding, as seen in the present review. Although a greater platelet response did not occur for the children in the two studies analyzed, a lower incidence of bleeding was demonstrated. This lack of global platelet response can possibly be explained by the small number of studies found (*n* = 2) and uncertainty related to their small samples. In the studies by Grainger et al. [23] and Bussel et al. [20], children showed a trend for better global platelet response. The platelet count was evaluated considering a cut-off point of 50,000/mm^3^; absolute values were not evaluated. Absolute platelet counts are related to quality of life (regarding physical activity, energy levels, and social life [17]); future studies in children analyzing the absolute platelet counts are necessary.

Our review is different from previous publications regarding separate evaluation of adults and children, the extensive search in the databases to find the evidence available on the topic, and a specific inclusion criterion focused on helping decision making in clinical practice. However, it also has some limitations. First, although we did all the searches in most of the major health databases, we cannot rule out the possibility that we may have missed a study that could been included in our review. Second, few studies were found recruiting children with ITP, and more studies in this population are needed. Finally, most of the studies were carried out in high-income developed countries, which restricts the results to this population. Further studies are needed in underdeveloped or middle-income countries, where access to this medication is restricted, to investigate whether the effectiveness of the results are reproducible. The clinical use of eltrombopag in refractory children must balance the risks and benefits. The persistence of severe thrombocytopenia implies a burden for society, a reduction in the quality of life of these patients, and potential losses of years of life or preventable fatalities in early life. In pediatric patients who do not respond to various types of treatment, the use of eltrombopag may be the wise choice for those with ITP in Brazil. Future longitudinal studies are needed to clarify these uncertainties in pediatric populations. In low-income countries, the ITP burden remains and thus is an important public health issue.

## 5. Conclusions

This meta-analysis demonstrated that eltrombopag was significantly effective for adults, with better platelet response, lower incidence of bleeding, and no difference in adverse events when compared with placebo. In children, despite a lower incidence of bleeding, eltrombopag did not show a significantly better platelet response, requiring further studies to obtain more robust results. Treatment with eltrombopag protected adults and children from severe disease and death.

## Figures and Tables

**Table 1:** Quality of evidence found for the use of eltrombopag compared with placebo in adults and children with immune thrombocytopenic purpura.

	Quality of the Study				Number of Events		Effect: RR ^4^ (95% CI)	Quality
	No. of Studies	Risk of Bias ^1^	Inconsistency ^2^	Imprecision ^3^	Intervention Group	Control Group		
**Platelets ≥ 50.000/mm^3^**								
Adults	7	No risk of serious bias	Without serious inconsistency	Without serious imprecision	308	40	3.65 (2.39–5.55)	⊗ ⊗ ⊗ High
Children	2	Without risk of serious bias	Serious inconsistency	Serious imprecision	53	8	3.93 (0.56–27.79)	⊗ Low
**Bleeding (WHO classification > 2)**								
Adults	6	Without risk of serious bias	Without serious inconsistency	Serious imprecision	106	67	0.80 (0.52–1.22)	⊗ ⊗ Moderate
Children	2	Without risk of serious bias	Without serious inconsistency	Serious imprecision	15	15	0.47 (0.27–0.83)	⊗ ⊗ Moderate
**Use of rescue therapy**								
Adults	5	Without risk of serious bias	Without serious inconsistency	Serious imprecision	57	72	0.40 (0.29–0.56)	⊗ ⊗ Moderate
Children	1	Without risk of serious bias	Without risk of serious inconsistency	Serious imprecision	28	7	1.96 (1.02–3.76)	⊗ ⊗ Moderate
**Lasting response**								
Adults	2	Without risk of serious bias	Serious inconsistency	Serious imprecision	82	13	3.17 (1.07–9.43)	⊗ Low
Children	1	Without risk of serious bias	Without serious inconsistency	Serious imprecision	13	1	6.36 (0.89–45.53)	⊗ ⊗ Moderate
**Adverse events**								
Adults	5	Without risk of serious bias	Without serious inconsistency	Serious imprecision	38	18	0.99 (0.55–1.78)	⊗ ⊗ Moderate
Children	2	Without risk of serious bias	Without serious inconsistency	Serious imprecision	10	8	0.59 (0.25–1.41)	⊗ ⊗ Moderate
**Platelets ≥ 50,000/mm^3^**								
Adults	7	Without risk of serious bias	Without risk of serious bias ^1^	Without serious imprecision	308	40	3.65 (2.39–5.55)	⊗ ⊗ ⊗ High
Children	2	Without risk of serious bias	Serious inconsistency	Serious imprecision	53	8	3.93 (0.56–27.79)	⊗ Low

CI, confidence interval; RR, relative risk; WHO, World Health Organization; ⊗, number of domains met ^1^ >25% of participants at high risk of bias. ^2^ Heterogeneity between studies was high (I^2^ > 50%). ^3^ Total events < 300 for each result. ^4^ Relative risk of eltrombopag compared with the placebo group.

## Data Availability

Not applicable.

## Supplementary Materials

The following supporting information can be downloaded at: https://www.mdpi.com/article/10.3390/jcm12123872/s1, Table S1. Characteristics of the studies included in the analysis.
