# Supplementary material for: Eltrombopag for Adults and Children with Immune-Refractory Thrombocytopenic Purpura: A Systematic Review
Source: J Clin Med. 2023 Jun 6;12(12):3872. doi: 10.3390/jcm12123872 (PMC10299452; doi:10.3390/jcm12123872)
Supplement: Supplementary file 1 [file jcm-12-03872-s001.zip › jcm-2344591-supplementary.pdf]

Journal of
Clinical Medicine

MDPI

Systematic Review

Eltrombopag for Adults and Children with Immune-Refractory Thrombocytopenic Purpura: A Systematic Review

Supplemental material

Search strategy

1. "immune thrombocytopenia".ab,ti,kw.
2. exp Purpura, Thrombocytopenic, Idiopathic/
3. exp Receptors, Thrombopoietin/ or Thrombopoiesis/ or Thrombopoietin/
4. thrombocytopeni*.ab,ti,kw.
5. "Thrombopoietin Receptors".ab,ti,kw.
6. "Thrombopoie*".ab,ti,kw.
7. Eltrombopag.ab,ti,kw. or Eltrombopag/
8. 1 or 2 or 3 or 4 or 5 or 6
9. (randomized or randomised).ab,ti.
10. placebo.ab,ti.
11. dt.fs.
12. randomly.ab,ti.
13. trial.ab,ti.
14. groups.ab,ti.
15. Animals/
16. Humans/
17. or/9-14
18. 15 not (15 and 16)
19. 17 not 18
20. 7 and 8 and 19

Table S1. Characteristics of the studies included in the analysis

Study	Country	Sample characteristic	Number of patients with <15,000/mm^3	Intervention/control group	Outcomes analyzed	Duration
Cheng et al., 2011 [22]	United States, Austria, Canada, China, Czech Republic, Denmark, Finland, France, Germany, Greece, India, Italy, Netherlands, New Zealand, Peru, Poland, Russian, Slovakia, Spain, Tunisia, Croatia, England and Vietnam	Total: 197 patients with ITP<br>Eltrombopag: *n* = 135; M, 42; F, 93; median age, 52.5 years (IQR, 43–63 years)<br>Placebo: *n* = 62; M, 19; F, 43; median age, 47.0 years (IQR, 34–56 years)	Placebo 30<br>Eltrombopag 67	Eltrombopag 50 mg/day; maximum dose, 75 mg/day (platelets <50 ×10^3/mm^3 after day 22)<br>Platelets >200 × 10^3/mm^3: 25 mg/day<br>Platelets >400 ×10^3/mm^3: suspend<br>Week 6: platelets >100 ×10^3/mm^3 in two consecutive weeks, reduce or discontinue treatment. Previous concomitant treatment<br>Placebo	Median platelet count<br>Clinically significant bleeding<br>Reduced or discontinued treatments<br>Rescue treatment	**6 months**
Bussel et al., 2015 [8]	22 centers in the US, UK, Canada,	Total: 67 patients	Placebo 11<br>Eltrombopag 23	Double blinded phase:	Platelet response for more than 6 weeks<br>Need for rescue therapy	**24 weeks**

Study	Country	Sample characteristic	Number of patients with <15,000/mm^3	Intervention/control group	Outcomes analyzed	Duration
	Spain, France, and the Netherlands	Eltrombopag: *n* = 45; M, 18; F, 27; median age, 9 years (IQR, 8–10 years)<br>Placebo: *n* = 22; M, 9; F, 13; median age, 10 years (IQR, 8–12 years)		12–17 years: 37.5 mg/day (initial dose)<br>6–11 years: ≥27 kg, 50 mg/day (25 mg Asians) ; <27 kg, 25 mg/day (12.5 mg Asians)<br>1–5 years: 1.5 mg/kg/day (0.8 mg/kg/day Asians)<br>Placebo: GlaxoSmithKline	Clinically significant bleeding (WHO grades 2–4)<br>Grade 3 or greater bleeding events<br>Grade 3 or 4 adverse events	
Bussel et al., 2009 [23]	23 centers: Germany, Greece, China, Korea, New Zealand, Pakistan, Poland, Romania, Russia, Slovenia, Thailand, England and Taiwan	Total: 114 patients<br>Eltrombopag: *n* = 76; M, 33; F, 43; median age, 47 years (IQR, 19–84 years)<br>Placebo: *n* = 38; M, 27; F, 11; median age, 51 years (IQR, 21–79 years)	Placebo 17<br>Eltrombopag 38	Eltrombopag (GlaxoSmithKline, Ware, UK) 50 mg/day<br>Platelets <50 × 10^3/mm^3 after 3 weeks: increase dose to 75 mg/day<br>Platelets >200 ×10^3/mm^3: discontinue<br>Placebo: GlaxoSmithKline for 6 weeks	Platelet counts: ≥50 × 10^3/mm^3 and at least twice the baseline value:<br>Eltrombopag: 42 (58%)<br>Placebo: 5 (14%)<br>Bleeding symptoms:<br>Eltrombopag: 20 (39%)<br>Placebo: 18 (60%)<br>Grade 3 or 4 adverse events:	6 weeks

Study	Country	Sample characteristic	Number of patients with <15,000/mm^3	Intervention/control group	Outcomes analyzed	Duration
					Eltrombopag: 2 (3%)<br>Placebo: 1 (3%)	
Bussel et al., 2007 [24]	44 centers: Germany, Greece, China, Korea, New Zealand, Pakistan, Poland, Romania, Russia, Slovenia, Thailand, England) between February and November 2005; at least 18 years of age	Total: 117 patients<br>Eltrombopag 30 mg: *n* = 30; M, 14; F, 16; median age, 51 years (IQR, 23–79 years)<br>Eltrombopag 50 mg: *n* = 30; M, 9; F, 21; median age, 45 years (IQR, 23–81 years)<br>Eltrombopag 75 mg: *n* = 28; M, 8; F, 20; median age, 55 years (IQR, 18–85 years)	Placebo 14<br>Eltrombopag 30 mg 15<br>Eltrombopag 50 mg 12<br>Eltrombopag 70 mg 15	Received placebo or eltrombopag 30, 50, or 75 mg<br>Withhold treatment if platelet count >200 × 10^9	Platelet count ≥50 × 10^3/mm^3:<br>Eltrombopag 75 mg: 21(81%)<br>Eltrombopag 50 mg: 19 (70%)<br>Eltrombopag 30 mg: 8 (28%)<br>Placebo: 3 (11%)<br>Incidence of bleeding events:<br>Eltrombopag 75 mg: 8 (4%)<br>Eltrombopag 50 mg: 2 (7%)<br>Eltrombopag 30 mg: 5 (17%)<br>Placebo: 4 (14%)<br>Adverse effects (grades 3 and 4)<br>Eltrombopag 75 mg: 3 (11%)<br>Eltrombopag 50 mg: 4(13%)<br>Eltrombopag 30 mg: 2 (7%)	6 weeks

Study	Country	Sample characteristic	Number of patients with <15,000/mm³	Intervention/control group	Outcomes analyzed	Duration
		Placebo: *n* = 29; M, 13; F, 16; median age, 42 years (IQR, 18–85 years)			Placebo: 4 (14%)	
Grainger et al., 2015 [9]	38 centers in 12 countries (Argentina, Czech Republic, Germany, China, Israel, Italy, Russia, Spain, Taiwan, Thailand, UK, and USA)	Total: 92 patients<br>Eltrombopag: *n* = 63; M, 33; F, 30; median age, 9.4 years (IQR, 8.2–10.5 years)<br>Placebo: *n* = 29; M, 15; F, 14; median age, 9.8 years (IQR, 8.3–11.3 years)	Placebo 19<br>Eltrombopag 38	Eltrombopag:<br>1–5 years: 1.2 mg/kg/day (0.8 mg/kg/day for East Asian patients)<br>6–11 years: ≥27 kg, started treatment at 50 mg/day (25 mg/day for East Asian patients); <27 kg, started treatment at 37.5 mg/day (25 mg/day for East Asian patients)<br>12–17 years: ≥27 kg, 50 mg/day (25 mg/day for East Asian patients); <27 kg, 37.5 mg/day (25 mg/day for East Asian patients<br>Placebo	Platelet count >50× 10^3/mm^3:<br>Eltrombopag 25 (40%)<br>Placebo 1 (3%)<br>Bleeding grades 2–4:<br>Eltrombopag 3 (5%)<br>Placebo 2 (7%)<br>Serious adverse events:<br>Eltrombopag 5 (8%)<br>Placebo 4 (14%)	24–28 weeks

Study	Country	Sample characteristic	Number of patients with <15,000/mm^3	Intervention/control group	Outcomes analyzed	Duration
**Liu et al., 2020 [25]**	China	Total: 150 patients<br>Eltrombopag: *n* = 100; M, 27, F, 73; median age, 45 years (SD 15.9 years)<br>Placebo: *n* = 50; M, 11; F, 38; median age, 40.9 years (SD, 12.65 years)<br>Placebo: *n* = 50; M, 11; F, 38; median age, 40.9 years (SD, 12.65 years)	Not specified	Eltrombopag: 25 mg/day (modified or suspended dose according to platelet value)<br>If platelets <50 × 10^9, increase the dose by 25 mg, reaching a maximum dose of 75 mg day<br>Placebo	Platelet count >50 × 10^3/mm^3:<br>Eltrombopag 38 (38%)<br>Placebo 10 (20%)<br>Grade 2 to 4 bleeding:<br>Eltrombopag 13 (13%)<br>Placebo 3 (6%)<br>Serious adverse events:<br>Eltrombopag 11 (11%)<br>Placebo 6 (12%)	**24 weeks**
**Yang et al., 2016 [26]**	China	Total: 155 patients<br>Eltrombopag: *n* = 104; M, 27 F, 77; median age, 48 years (IQR, 18–84 years)	Placebo 28<br>Eltrombopag 54	Eltrombopag: 25 mg for 8 weeks (maximum 75 mg day)<br>Placebo<br>Stage II: Patients who received eltrombopag in Stage I continued on the dose	Platelet count >50 × 10^3/mm^3:<br>Eltrombopag 60 (57.7%)<br>Placebo 3 (6%)<br>WHO grade 2-4 bleeding:<br>Eltrombopag 14 (13.5%)<br>Placebo 6 (11.5%)	**8 weeks**

Study	Country	Sample characteristic	Number of patients with <15,000/mm^3	Intervention/control group	Outcomes analyzed	Duration
		Placebo: n = 51; M, 11; F, 40; median age, 42 years (IQR, 22–66 years)		administered at the end of stage I, patients who received placebo in stage I started active treatment with an initial dose of 25 mg of eltrombopag day	Serious adverse events:<br>Eltrombopag 5 (4.8%)<br>Placebo 5 (9.8%)	
**Tomiyama et al., 2012 [27]**	Japan	Total: 23 patients<br>Eltrombopag: n = 15; M, 7 F, 8; median age, 58 years (IQR, 26–72 years)<br>Placebo n = 8; M,1 F,7; Median age 60.5 years (IQR:38-72)	Placebo 6<br>Eltrombopag 3	Phase 1: double-blind, randomized, placebo-controlled phase of 6 weeks<br>Phase 2: open label for 6 weeks<br>Eltrombopag 12.5 mg/day (maximum dose 50 mg/day)<br>Placebo 25 mg/day	Platelet count >50× 10^3/mm^3:<br>Eltrombopag 9 (60%)<br>Placebo 0 (0%)<br>Any adverse events:<br>Eltrombopag 11 (6%)<br>Placebo 2 (1%)	**26 weeks**
**Huang et al., 2018 [28]**	**China**	**Total: 35 patients ≥18 years**	**Placebo 6**<br>**Eltrombopag 3**	**If platelets <50 × 10^9/L: increase dose 25 mg (with a maximum of 75 mg) platelets (150–250) × 10^9/L: reduce to the nearest**	**Platelet count >50× 10^3/mm^3:**<br>**Eltrombopag 11 (64%)**<br>**Placebo 2 (11.1%)**<br>**Use of rescue medication:**	**6 weeks**

Study	Country	Sample characteristic	Number of patients with <15,000/mm3	Intervention/control group	Outcomes analyzed	Duration
		**Eltrombopag**: ***n*** **= 17; M, 2**; **F, 15; median age**, **50 years (IQR**, **24 62** years**)** **Placebo**: ***n*** **= 18; M, 4**; **F, 14; median age**, **39.5 years (IQR**, **22–66** years**); 25 mg/day**		**lower dose (example: 50 mg/day to 25 mg/day), or reduce the frequency of** the **dose (example: 25 mg/day to 25 mg once every 2 days**). If **platelets >250 × 10⁹/L**, **stop the drug and** resume **taking the drug after platelets <100 × 10⁹/L**	**Eltrombopag 0 (0%)** **Placebo 8 (44.4%)** **Adverse events**: b**leeding:** **Eltrombopag 0 (0%)** **Placebo 4 (22.2%)**	

ITP, immune thrombocytopenic purpura; M, male; F, female; IQR, interquartile range.
